# Supplementary material for: Out-of-pocket payments and associated factors among hypertensive patients insured under the National Health Insurance Scheme in a referral hospital, Ghana
Source: PLoS One. 2026 May 14;21(5):e0348971. doi: 10.1371/journal.pone.0348971 (PMC13175461; doi:10.1371/journal.pone.0348971)
Supplement: Attachment — Submitted filename: Dear author.docx [file pone.0348971.s002.docx]

Dear author, thank you for your manuscript.

The following comments are sent to enrich your work

1. It is better to focus more on the main challenge in the introduction and avoid additional explanations. State the main reason for choosing this topic and its importance.
2. State the time frame of the research.
3. Abbreviation for out-of-pocket payments is OOP not OPP.
4. The use of a cross-sectional method may not be able to show the cause-and-effect relationship between factors related to out-of-pocket payments. This type of study only examines the current situation and may ignore changes over time
5. This article does not provide a precise definition of "out-of-pocket payments" and does not distinguish between authorized and unauthorized payments. This could lead to misinterpretation of the data.
6. The sample was drawn from only three regions, which may not be representative of the entire country. This makes it difficult to generalize the results to other regions of Ghana.
7. The article does not fully assess the impact of out-of-pocket payments on the economic situation of households, especially poor households, which is essential to achieving the Sustainable Development Goals.
8. The article does not provide a precise definition of “out-of-pocket payments” and does not distinguish between authorized and unauthorized payments. This can lead to misinterpretation of the data.
9. Although the article provides recommendations, it does not provide practical solutions for implementing these recommendations.
