# Supplementary material for: Out-of-pocket payments and associated factors among hypertensive patients insured under the National Health Insurance Scheme in a referral hospital, Ghana
Source: PLoS One. 2026 May 14;21(5):e0348971. doi: 10.1371/journal.pone.0348971 (PMC13175461; doi:10.1371/journal.pone.0348971)
Supplement: Attachment — Submitted filename: Response to Reviewers.docx [file pone.0348971.s004.docx]

University of Health and Allied Sciences

Fred N. Binka School of Public Health

PMB 31, Ho

May 28, 2025

The Editor-in-Chief

Plos One

Dear Dr. Camps,

**Subject: Response to Reviewers for Manuscript [PONE-D-25-08305]**

I sincerely appreciate the time and effort that you and the reviewer have invested in evaluating our manuscript, titled “Out-of-pocket payments and associated factors among hypertensive patients insured under the National Health Insurance Scheme in a referral hospital, Ghana”. We are grateful for the constructive feedback, which has helped us improve the quality and clarity of our work.

Below, we provide detailed responses to the editor and reviewer’s comments. We have carefully revised the manuscript to address all concerns and believe that the updated version strengthens our study.

**Journal requirements:**

1. Please ensure that your manuscript meets PLOS ONE's style requirements, including those for file naming.

Response: Thank you for pointing this out. We have thoroughly reviewed the journal’s submission guidelines and revised the paper accordingly. This includes adjustments to section headings, line spacing, abstract format and other formatting specifications.

2. We note that Figure 1 in your submission contain [map/satellite] images which may be copyrighted. We require you to either (1) present written permission from the copyright holder to publish these figures specifically under the CC BY 4.0 license, or (2) remove the figures from your submission.

Response: Thank you for pointing this out. We have removed the figure from the paper to avoid any copyright infringement.

**Reviewer #1 comments:**

1. *Comment:* It is better to focus more on the main challenge in the introduction and avoid additional explanations. State the main reason for choosing this topic and its importance.

*Response:* Thank you for this insightful comment. We appreciate your points on the need to be concise and specific. To improve clarity and focus, we have made major edits for conciseness while retaining key references which directly support the rationale for the research. We believe that this revision strengthens your reading experience by leading to the main reason for choosing this topic and its importance more quickly.

2. *Comment:* State the time frame of the research.

*Response:* Thank you for pointing this out. We have revised the manuscript to include the time frame of the research (See Pages 2, 7 & Lines 25, 116)

3. *Comment:* Abbreviation for out-of-pocket payments is OOP not OPP.

*Response:* Thank you for noting this. We have revised the entire manuscript to reflect OOP as the appropriate abbreviation for out-of-pocket and not OPP.

4. *Comment:* The use of a cross-sectional method may not be able to show the cause-and-effect relationship between factors related to out-of-pocket payments. This type of study only examines the current situation and may ignore changes over time.

*Response:* Thank you for this important point. We agree that cross-sectional studies are not able to determine causality and do not consider changes overtime. We have therefore acknowledged that our adoption of this study design is a key limitation of our study. This acknowledgement enhances the integrity of the paper.

5. *Comment:* This article does not provide a precise definition of "out-of-pocket payments" and does not distinguish between authorized and unauthorized payments. This could lead to misinterpretation of the data.

*Response:* Thank you for this insightful comment. We agree that the lack of a clear distinction between authorized and unauthorized out-of-pocket payments may lead to a misrepresentation of our findings. We have therefore acknowledged this as an important limitation of our study. This admission strengthens the transparency and credibility of the paper.

6. *Comment:* The sample was drawn from only three regions, which may not be representative of the entire country. This makes it difficult to generalize the results to other regions of Ghana.

*Response:* Thank you for this insightful comment. We agree that our study may not be generalizable to the other regions of Ghana. As such, we have acknowledged this as a major limitation of our study.

7. *Comment:* The article does not fully assess the impact of out-of-pocket payments on the economic situation of households, especially poor households, which is essential to achieving the Sustainable Development Goals.

*Response:* Thank you for this important point. We agree that our study did not comprehensively evaluate the impact of out-of-pocket payments on the economic situation of households. We have therefore acknowledged this as a key limitation of our study which we hope strengthens the transparency of the paper.

8. *Comment:* Although the article provides recommendations, it does not provide practical solutions for implementing these recommendations.

*Response:* Thank you for noting this. We have expanded our recommendations to include the key stakeholders responsible for implementing the recommendations we gave.

We hope that these revisions satisfactorily address the editor and reviewer’s concerns. We appreciate the opportunity to improve our manuscript and look forward to your feedback.

Thank you for your time and consideration.

Sincerely,

**Angela Nana Esi Ackon**

[**angela.aan2009@gmail.com**](mailto:angela.aan2009@gmail.com)
