# Supplementary material for: Out-of-pocket payments and associated factors among hypertensive patients insured under the National Health Insurance Scheme in a referral hospital, Ghana
Source: PLoS One. 2026 May 14;21(5):e0348971. doi: 10.1371/journal.pone.0348971 (PMC13175461; doi:10.1371/journal.pone.0348971)
Supplement: Attachment — Submitted filename: Response_to_Reviewers_auresp_2.docx [file pone.0348971.s005.docx]

University of Health and Allied Sciences

Fred N. Binka School of Public Health

PMB 31, Ho

April 13, 2026

The Academic Editor

Plos One

Dear Professor Ezenduka,

**Subject: Response to Reviewers for Manuscript [PONE-D-25-08305R1]**

I sincerely appreciate the time and effort that you and the reviewers have invested in evaluating our manuscript, titled “Out-of-pocket payments and associated factors among hypertensive patients insured under the National Health Insurance Scheme in a referral hospital, Ghana”. We are grateful for the constructive feedback, which has helped us improve the quality and clarity of our work.

Below, we provide detailed responses to the editor and reviewers’ comments. We have carefully revised the manuscript to address all concerns and believe that the updated version strengthens our study.

Reviewer #1: Dear Editor,
Hello
I confirm that all the reviewer comments provided to the authors have been fully addressed and incorporated into the revised version of the manuscript. The authors have responded appropriately to each point, and the revisions made adequately reflect the feedback previously given.

Reviewer #2: The revised manuscript provides a timely and policy-relevant analysis of out-of-pocket payments among insured hypertensive patients in Ghana, addressing an important gap in the universal health coverage discourse. The methodology is appropriate, with clear use of cross-sectional design, adequate sample size, and robust multivariable logistic regression analysis. The findings are clearly presented and demonstrate significant associations with meaningful public health implications. The discussion appropriately contextualizes the results within SDG targets and national insurance policy objectives. I recommend acceptance of the manuscript in its current form.

Reviewer #3: General comment
The authors have clearly engaged with the prior reviewer comments and have added explicit limitations, clarified timeframe, corrected terminology, and expanded recommendations. The manuscript is scientifically coherent and addresses an important health-financing question. However, several editorially critical and methodologically relevant issues remain unresolved, including internal inconsistencies, reporting clarity, sampling description accuracy, and analytical interpretation.
Specific comments

1. ***Comment:*** While the authors state that all instances of OPP were replaced with OOP, the revised manuscript still contains multiple residual “OPPsOOP” artifacts, indicating incomplete editing and typesetting inconsistencies (visible in conclusions and limitations sections).

***Response:*** Thank you for pointing this out. We have thoroughly revised the entire manuscript and expunged all residual artifacts. We have also ensured consistency in editing across all sections.

2. ***Comment:*** The limitation about not distinguishing authorised vs unauthorised payments is acknowledged, which is good, but the manuscript still does not operationally define how respondents interpreted OOP payments during data collection. This raises interpretability concerns about whether co-payments and informal payments, alongside drug purchases outside facility were included. In essence, the limitation statement remains insufficient as readers need clearer operational framing in the Methods.

***Response:*** Thank you for this insightful comment. We have revised the methods to clearly detail how respondents were instructed to interpret payments. Our study however excludes payments made outside of the facility since we sought to examine the out-of-pocket payments made which would have ordinarily been covered by the NHIS. In our revision, we have strengthened the operational definition of payments in the methods section. We believe that these revisions would enhance transparency (See Page 9, Lines 164-173).

3. ***Comment:*** While the limitation about single-region sampling is properly acknowledged, the reviewer response incorrectly states the sample was from three regions whereas the manuscript clearly describes one facility in the Volta Region. This mismatch should be corrected for consistency.

***Response:*** Thank you for this important observation. We acknowledge that our sample was drawn from one facility in the Volta region and hence our response to the previous review comment should have clarified the absence of any mention of three regions in our manuscript.

4. ***Comment:*** While stakeholder identification has been added to the recommendations, most of the policy recommendations remain generic, with limited linkage to study findings. I recommend that the authors tie each recommendation directly to a specific empirical result.

***Response:*** Thank you for this insightful comment. We have revised the recommendations to relate it more closely with the results to enhance their practical relevance.

5. ***Comment:*** The manuscript states: sample size target = 422 (after non-response adjustment), and actual sample recruited = 389. However, there is no explanation for this discrepancy. This reporting gap could affect reproducibility and transparency. In addition, the randomisation description suggests systematic daily recruitment rather than true simple random sampling, and while a potential repeat-visit bias mitigation is described, it was not validated.

***Response:*** Thank you for this insightful comment. We have revised the methods to clarify that the discrepancy reflects practical recruitment constraints rather than methodological issues. We however successfully recruited 393 respondents but 4 were not included in the analysis due to missing data. Also, we have now revised the methods section to clarify the process of selecting participants. Regarding the mitigation of potential repeat-visit bias, we agree that our initial description lacked validation and we have revised our limitations to clearly acknowledged this limitation. We believe these revisions strengthen methodological clarity of the manuscript.

6. ***Comment:*** While the authors acknowledge that outcome variables rely entirely on self-report, this could be better contextualised along recall bias, misclassification and social desirability bias risks.

***Response:*** Thank you for this observation. We have revised the limitations to address the possibilities of recall bias, misclassifications and social desirability bias. We have also clarified in our methods and limitations sections and provided a fuller account of both the risks and the mitigation strategies employed. Firstly, that questionnaires were administered as exit interviews, immediately after respondents received treatment which reduced the risk of recall bias. Again, we have noted that we tried to reduce misclassification by providing detailed breakdown of possible health services that may have resulted in payments in the questionnaire and with regards to social desirability bias, we minimised this by conducting the interviews privately.

7. ***Comment:*** While the Logistic regression seems appropriate, some missing essentials include variable selection criteria, model diagnostics, multicollinearity checks, and sensitivity analysis. These elements of model transparency would improve rigour. ***Response:*** Thank you for this important comment. We could not conduct sensitivity analysis due to the distribution of the outcome variables which limited the potential for meaningful variation across alternative model specifications. We have expanded our limitations to acknowledge this (See Page 27, Lines 494-495).
8. ***Comment:*** In result interpretation, the prevalence estimate (97.2%) is extremely high relative to cited Ghanaian literature. While the discussion attributes this to sample homogeneity, this explanation is not empirically demonstrated. Thus, the conclusion risks overstating generalisable burden.

***Response:*** Thank you for this important comment. We have made revisions in our manuscript to ensure that we remain within the limits of our evidence. We have thus accurately presented results, expanded the limitations to fully explain methodological and contextual constraints and revised our conclusions to avoid overstating implications to enhance the credibility of the manuscript.

9. ***Comment:*** Several issues require correction as multiple sections contain duplicated or corrupted terms (e.g., OPPsOOP). For instance, percent denominators are sometimes unclear, there are overlapping categories, and Fisher vs Chi-square labelling seem inconsistent.

***Response:*** Thank you for this insightful comment. We have revised the manuscript and corrected all instances of duplication or corrupted text. We have clarified the denominators used for each percentage, ensuring consistency and transparency across tables and narrative results. These corrections improve clarity, accuracy, and consistency throughout the manuscript.

10. ***Comment:*** Also, there are some logical phrasing issues such as “findings are important and generalisable”. This is contradicted by the already stated sampling limitations. Again, the claim that findings are generalisable is not sufficiently justified given the single facility, region-specific context, and older patient skew. A revision should reflect analytic relevance, not population generalisability.

***Response:*** Thank you for this important observation. We have made major revisions in our manuscript to ensure that we avoid overstating implications of our findings. We believe this would enhance the credibility of the manuscript.

11. ***Comment:*** The revised manuscript does not report any formal robustness or sensitivity analyses beyond the primary logistic regression model. Including some basic model diagnostics or alternative specifications would strengthen confidence in the findings. Given the very high prevalence of OOP payments and reliance on self-reported measures, demonstrating that the main associations are not sensitive to modelling choices would enhance the methodological transparency and credibility of the results.

***Response:*** Thank you for this important observation. Because our study reported a very high prevalence of OOP payments, the scope for meaningful sensitivity analysis was limited given that for such a skewed distribution, sensitivity analyses across subsamples or alternative specifications would not materially change the results, as the imbalance in outcome frequencies constrains model variation. We have acknowledged this in our limitations and noted that future research could consider alternative approaches if outcomes with greater variability are available.

We hope that these revisions satisfactorily address the editor and reviewers’ concerns. We appreciate the opportunity to improve our manuscript and look forward to your feedback.

Thank you for your time and consideration.

Sincerely,

**Angela Nana Esi Ackon**

[**angela.aan2009@gmail.com**](mailto:angela.aan2009@gmail.com)
